# An investigation of the control of quadriceps in people who are hypermobile; a case control design. Do the results impact our choice of exercise for people with symptomatic hypermobility?

**DOI:** 10.1186/s12891-022-05540-1

**Published:** 2022-06-23

**Authors:** Michael Long, Louise Kiru, Jamila Kassam, Paul H. Strutton, Caroline M. Alexander

**Affiliations:** 1 MSk Lab, Department of Surgery and Cancer, Imperial College London, London, UK; 2 Department of Therapies, Charing Cross Hospital, Imperial College Healthcare NHS Trust, Fulham Palace Road, London, W6 8RF UK

**Keywords:** Hypermobility, Hoffman's reflex, Transcranial magnetic stimulation, Electromyography, Proprioception

## Abstract

**Background:**

People with symptomatic hypermobility have altered proprioception however, the origin of this is unclear and needs further investigation to target rehabilitation appropriately. The objective of this investigation was to explore the corticospinal and reflex control of quadriceps and see if it differed between three groups of people: those who have symptomatic hypermobility, asymptomatic hypermobility and normal flexibility.

**Methods:**

Using Transcranial Magnetic Stimulation (TMS) and electrical stimulation of peripheral nerves, motor evoked potentials (MEPs) and Hoffman (H) reflexes of quadriceps were evoked in the three groups of people. The threshold and latency of MEPs and the slope of the input–output curves and the amplitude of MEPs and H reflexes were compared across the groups.

**Results:**

The slope of the input–output curve created from MEPs as a result of TMS was steeper in people with symptomatic hypermobility when compared to asymptomatic and normally flexible people (*p* = 0.04). There were no other differences between the groups.

**Conclusion:**

Corticospinal excitability and the excitability at the motoneurone pool are not likely candidates for the origin of proprioceptive loss in people with symptomatic hypermobility. This is discussed in the light of other work to suggest the receptor sitting in hypermobile connective tissue is a likely candidate. This suggests that treatment aimed at improving receptor responsiveness through increasing muscle tone, may be an effective rehabilitation strategy.

## Introduction

People with a particularly large degree of joint range of motion are classified along a spectrum. At one end, Asymptomatic Generalised Joint Hypermobility (GJH) is characterised by excessive motion without musculoskeletal problems and is classified using the validated Beighton score [1–3]. This is a score out of nine for excessive joint motion such as hyperextending elbows and knees. In contrast, at the other end of the spectrum is Hypermobility Spectrum Disorder (HSD) and hypermobile Ehlers Danlos Syndrome (hEDS) – together previously termed Joint Hypermobility Syndrome (JHS; [4, 5]. These are characterised by many symptoms that include dyskinesia and pain [6, 7]. Symptomatic hypermobility is an inherited connective tissue disorder [4, 8] with additional disabling characteristics such as muscle weakness [9–11], easily provoked soft-tissue injury [12–14], varicose veins, uterine or rectal prolapse and hernias [15–18], anxiety and fatigue [16, 18–23]. These symptoms impact function and quality of life [11, 24, 25]. Pain has been reported to be most commonly felt in the knees [26] and spine [27] but usually occurs in multiple joints.

Little focus has been paid to understanding the symptomatic condition [17, 28]. However, although the prevalence in the general population is low, the number presenting to musculoskeletal services for treatment is high [29–32]. This high prevalence within musculoskeletal healthcare together with the impact of the condition, suggests that greater focus should be directed at understanding why hypermobility can be symptomatic, which could reveal targets for treatment.

The evidence base to support treatment design is still evolving [33–36]. However, guidelines and suggestions for treatment are limited by the underpinning literature [37, 38]. What is clear is that people with JHS test weak [9–11] and therefore physical therapies include strengthening muscles. However, impaired mechanisms of control are also thought important as they are believed to contribute to impairments in balance [39, 40], joint position sense [41–43] and reduced activity levels [18, 25]. Further, reported changes to proprioception and other mechanisms of control lead to speculation that they contribute to changes in kinetics and kinematics [7, 40, 44, 45] and poor joint stability resulting in minor but repetitive trauma [42, 46], which consequently could relate to the pain. Therefore, treatments also include the training of balance, joint proprioception [47, 48] and reaction to perturbations [49]. Although change to joint re position sense, perception of joint movement and muscle activity in response to perturbations have been widely investigated [18, 40, 41, 43, 46, 47, 50–52], we still do not understand if the issue relates to dysfunction of the central nervous system and/or dysfunction of the receptor sitting in hypermobile connective tissue. This is because the investigations to date incorporate an examination of the whole of the neural pathway from receptor to muscle, that includes transcortical pathways [41, 42, 47, 50]. However, the question of whether the central control differs and/or the responsiveness of the receptor sitting in hypermobile tissue is important. This is because if central control differs in people with symptomatic hypermobility then exercises related to challenging control can be designed within a package of treatment. However, if the problem with proprioception is due to receptors sitting in slack connective tissue [53, 54], such as muscle spindles sitting within lax musculature, then building muscle tone may be a more appropriate treatment strategy. We therefore still need to understand if the central nervous system excitability differs in this population.

Investigating central control through corticospinal and spinal reflex excitability in humans has long been established [55, 56]. Indeed, evoking spinal reflexes with electrical stimulation of peripheral nerves—the Hoffmann (H) reflex—has been used since the middle of the last century to investigate spinal excitability [56]. Methods of investigation of corticospinal control have been established since the 1980s using transcranial magnetic stimulation (TMS) to induce motor evoked potentials (MEPs, [55]). Taken together the amplitude of the H reflex and slope of its recruitment curve as well as the latency, threshold and input–output relationship of MEPs can give an understanding of the conduction and excitability of both the motoneurone pool and corticospinal pathway [57]. These long-established methods can be used to explore control in people with hypermobility.

Therefore, the aim of this study was to investigate whether there are differences to corticospinal and spinal excitability in people with JHS compared to people who are equally flexible but asymptomatic, and people who have normal flexibility. Our hypothesis was that people with symptomatic hypermobility would have reduced excitability of corticospinal and reflex control in comparison to people with asymptomatic hypermobility and people with normal flexibility.

## Materials and Method

### Participant selection

All experimental protocols were approved by National Research Ethics Service Committee London – Harrow (12/LO/1756). Participants were recruited from patients, staff and students from a large hospital and university, as well as in response to adverts directed towards members of the symptomatic hypermobile community (Ehlers-Danlos UK and Hypermobility Syndromes Association) and running clubs (eg ParkRun UK). With written and informed consent, three groups of participants who were 18 years or older were recruited. The three groups were healthy people with a Beighton score of 3 or less out of 9 (Normal Flexibility, no knee pain (NF)); people with a Beighton score of 4 or more but not fulfilling the Brighton criteria (Generalised Joint Hypermobility, no knee pain (GJH)) and finally people classified using the Brighton Criteria who complained of anterior knee pain (Joint Hypermobility Syndrome, plus knee pain (JHS)). The Brighton criteria classifies hypermobile people on the basis of major and minor criteria. Major criteria include joint pain for longer than 3 months in four or more joints along with a Beighton score of 4 or more out of 9. Minor criteria include joint dislocations, hyperextensibility of skin and hernias. People were classified as having JHS if they scored 2 major criteria or 1 major and 2 minor criteria or 4 minor criteria [21]. Quadriceps control was explored here as people with JHS commonly have knee symptoms [26], which is likely to impact quadriceps function. These classification criteria were chosen as the data collection began before the publication of new criteria for hEDS and HSD and in addition, this classification criteria have been useful when distinguishing between symptomatic and asymptomatic hypermobile people [11, 45]. Participants were excluded if they had any neurological disease or any medical illness unrelated to their hypermobility such as Rheumatoid Arthritis or previous fractures. In addition, people were excluded from the TMS study if they had history of head trauma with concussion or associated loss of consciousness, any neurological problems that include fainting, epilepsy, convulsions and seizures, metal implants such as surgical clips, implanted neurostimulators, cochlear implants or pacemaker; as well as currently taking any neuromodulatory medication such as amitriptyline or gabapentin [58].

As this work had not been done before, a sample size calculation was initially based upon MEP threshold differences between patients with another musculoskeletal problem, low back pain, and a healthy cohort. With an approximate 10% difference in threshold and 10% standard deviation, this indicated that 20 people in each group would be required to have 80% power at 5% significance. The sample size calculation was repeated during an interim analysis using our initial data after recruitment of 36 participants, and with an 8% difference in threshold and 10% standard deviation, this suggested a need to increase the sample size to 30 people in each group [59].

Baseline characteristics including Beighton score, age, ethnicity and sex were recorded. As previously mentioned, the Beighton score is a score out of nine for excessive joint motion such as hyperextending elbows and knees along with ability to touch the floor with the flattened hands whilst maintaining knee extension and hyperflexibility of the thumb and 5^th^ finger. If they had knee pain they were asked to complete a visual analogue scale (VAS; 0 cm to 100 cm scale) to record their current knee pain intensity. In order to understand the level of activity of participants, all participants were asked to complete The Human Activity Profile [60]. The Human Activity Profile lists 94 activities ranging from “Getting in and out of chairs or bed (without assistance)” to “Running or jogging 3 miles (4.8 km) in 30 min or less”. The participant is asked to mark whether they are still doing this activity, have stopped doing the activity or have never done the activity. The number of activities that the participant has stopped doing below their maximum activity level is subtracted from their maximum activity level to give an Adjusted Activity Score. Leg dominance was determined using the test outlined in Vauhnik et al. [61].

Participants sat on an adjustable height bed with their hips at 45 degrees of flexion and the knee in 35 degrees of flexion with the thigh resting over a specific support. Surface electromyography was used to record muscle activity of the Rectus Femoris (RF) on the dominant leg of people with NF and GJH who did not have knee pain or the most painful side of participants with JHS. Disposable single-use silver/silver chloride self-adhesive electrodes (Blue Sensor Q, Ambu) were placed on the dominant or painful leg. The electrodes were positioned on the skin halfway between the anterior superior iliac spine and the base of the patella [62]. Electrode orientation was such that they were parallel to the muscle fibres with an inter-electrode distance of 20 mm. The signal was amplified (NL844, Digitimer) and filtered (NL125/NL126, Digitimer) with a high pass of 6 kHz and low pass of 30 Hz. Data were sampled at 4000 Hz using a Micro 1401 analogue to digital converter (Cambridge Electronic Design [CED] Ltd) and collected using a PC with Signal software (Version 3.13, CED Ltd).

### Corticospinal investigation

To lower the threshold to evoke a Rectus Femoris (RF) MEP [63], participants undertook a maximum voluntary isometric contraction (MVC) and were then required to maintain 20% of the EMG generated at MVC during data collection by extending their knee. This was maintained using EMG biofeedback. TMS was applied to the hemisphere contralateral to the dominant or most painful side using a double 70 mm figure of eight coil (Bisim^2^, Magstim). The site for stimulation of the quadriceps motor cortex (hotspot) was found by moving the coil, positioned with the current delivered in an anterior–posterior orientation and tangentially to the skull at a 45 degree angle until the largest quadriceps MEP was elicited with the lowest stimulus intensity. The TMS was triggered every 5 s by the analogue to digital convertor.

Participants’ active motor threshold (AMT) was then calculated by reducing the stimulation intensity until 3 out of 6 stimulations evoked an MEP above the ongoing activity [64]. Stimulus intensity was then increased to 120% AMT and 5 stimuli were delivered. This enabled the intensity at which MEP latency was measured to be normalised between participants. Finally in order to obtain the MEP recruitment curve, participant’s EMG activity was recorded in response to stimulation at an intensity of 10% below AMT. The stimulus intensity was then increased in 10% increments until output was 100% of maximum stimulator output (%MSO). For those participants whose AMT was 70% or higher, MEPs were recorded in 5% increments until output reached 100% MSO. At each percentage increment, 3 stimuli were delivered.

### Reflex investigation

The anode was taped to the skin below the inguinal line on the superior anterior thigh on the side to be tested. A 1 ms square wave pulse was delivered using a constant current stimulator (DS7A, Digitimer) through a handheld roving cathode. The stimulus was triggered by the analogue to digital converter with an inter-stimulus interval of 5 s. The cathode was positioned on the skin over the femoral triangle until a motor (M) response was visualised. The stimulation intensity was adjusted until the amplitude of the M response plateaued, signifying maximal motor response (Mmax). Once the stimulus intensity to generate Mmax had been established it was lowered until subthreshold for both the M response and H reflex. The stimulus intensity was then increased in 0.5 mA increments every 3 stimuli until either the H reflex was no longer visible or the Mmax was attained.

### Data analysis

The H reflex amplitude and the MEP amplitude were both normalised to Mmax. The normalised H reflex and MEP amplitudes were then averaged at each stimulus intensity. These values were used to construct recruitment curves plotting the stimulus intensity against the amplitude of the response. As the M response and the H reflex of quadriceps were not always sufficiently separated in time, it was not possible to accurately determine the latencies of many H reflexes, therefore, these were not reported.

Where appropriate, the data were tested for normality (Shapiro–Wilk). Sex and ethnicity were compared across groups using the Chi Squared test. The age, Beighton score, activity levels, the slope of the upward portion of the normalised recruitment curves, the Hmax/Mmax, the MEPmax/Mmax, the threshold of the MEP and the latency of the MEP at 120% of active motor threshold were then compared across the three groups using a one way ANOVA (with Bonferroni correction for post hoc tests) or the equivalent tests for non-normally distributed data (Kruskal–Wallis One Way Analysis of Variance on Ranks with Dunn’s post hoc test; SigmaPlot statistical package; Version 11.0, Systat Software Inc.). Differences were considered significant when the *p* value was ≤ 0.05. All values are reported as means ± SD unless otherwise stated.

## Results

Ninety people were recruited; their demographics are detailed in Tables 1 and 2. Taken together, the demographics tended to match the common differences between such groups [11], with the people with normal flexibility having a lower Beighton score than the two hypermobile groups (*p* < 0.001), the proportion of women being lower in the NF (*p* < 0.02) and the JHS group being less active compared to the other two groups (*p* < 0.001). The ethnic origin of participants varied within each group, which was similar across the groups (Table 2; *p* = 0.89).

**Table 1:** Demographic details for three groups of participants

	NF (*n* = 30)	GJH (*n* = 30)	JHS (*n *= 30)	*p* value
**Age**	27.6 ± 6.3	27.1 ± 6.2	30.7 ± 8.9	0.18
**Sex**	63.3^a^	80.0	93.0	0.02
**Beighton**	1.1 ± 0.9^a^	5.9 ± 1.5	5.9 ± 1.7	0.001
**HAP (AAS)**	90.5 ± 8.5	87.1 ± 14.8	69.5 ± 20.8^a^	0.001

People with Normal flexibility, no knee pain (NF), Generalised Joint Hypermobility, no knee pain (GJH) and Joint Hypermobility Syndrome, plus knee pain (JHS). The percentage of females in the groups as well as the mean ± standard deviation of age, Beighton Score and Human Activity Profile (HAP; Adjusted Activity Score (AAS)) are given

^a^denotes a significant difference relative to the other two groups

**Table 2:** Ethnic origin of participants with Normal flexibility, no knee pain (NF), Generalised Joint Hypermobility, no knee pain (GJH) and Joint Hypermobility Syndrome, plus knee pain (JHS). The number of participants is given. There was no significant difference across the groups (p = 0.89)

	NF	GJH	JHS
**White British**	19	15	16
**Other White**	6	7	6
**Black/Black British**	0	1	0
**Asian/Asian British**	4	4	3
**Mixed**	0	1	3
**Other ethnic group**	1	1	1
**Participant chose not to record**	0	1	1

The mean (± standard deviation) severity of pain in the knee reported using the VAS by the JHS group was 42.4 mm ± 25.1 mm.

All recruited participants undertook the electrical stimulation of the femoral nerve. However, 11 participants were excluded from stimulation of the motor cortex. This was due to taking neuromodulatory drugs and/or a history of fainting (1 excluded from the GJH group and 10 excluded from the JHS group). This left 30 participants in the normal flexibility group, 29 in the GJH group and 20 in the JHS group who underwent TMS.

**Fig. 1:**
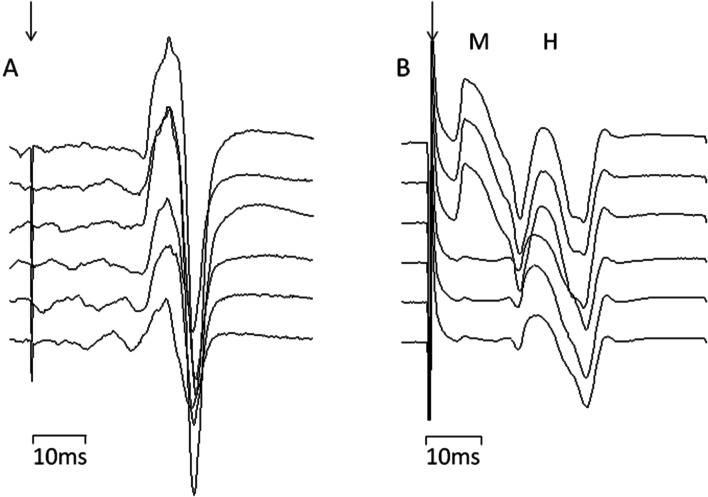
Averaged evoked responses from transcranial magnetic stimulation of the quadriceps motor cortex (**A**) evoking MEPs, and stimulation of the femoral nerve (**B**) evoking the Motor (M) response and Hoffman (H) reflex. The average responses increase in amplitude with an increase in stimulus intensity with the intensity increasing from the bottom trace to the top. The downward arrows mark the stimuli

Typical evoked responses are illustrated in Fig. 1, which illustrates the increase in MEP amplitude with increasing stimulus intensity (Fig. 1A); and the increase in H reflex amplitude with the M response evoked at a higher stimulus intensity than the H reflex (Fig. 1B).

### Corticospinal responses

**Fig. 2:**
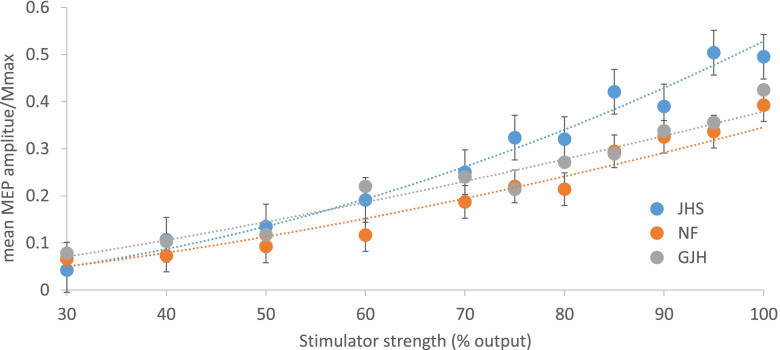
A grand average of the mean MEP amplitude as a proportion of Mmax against the Transcranial Magnetic Stimulator percentage output (%) for the three populations of people with Joint Hypermobility Syndrome, plus knee pain (JHS in blue), Generalised Joint Hypermobility, no knee pain (GJH in grey) and normal flexibility, no knee pain (NF in orange). Error bars represent the standard deviation. A line of best fit is added for each group

The slope of the MEP recruitment curve differed between groups (*p* = 0.04). The slope of the recruitment curve for the JHS group was steeper than those of the other two groups, which did not differ (see Fig. 2).

This suggests greater quadriceps corticospinal excitability in the JHS group. The MEPmax/Mmax (*p* = 0.54), MEP latency (*p* = 0.33) and threshold (*p* = 0.77) did not differ between groups. See Table 3 for the details of the results.

**Table 3:** Mean ± standard deviation of Motor Evoked Potential (MEP) data from transcranial magnetic stimulation for three groups of participants; people with Normal Flexibility, no knee pain (NF), Generalised Joint Hypermobility, no knee pain (GJH) and Joint Hypermobility Syndrome, plus knee pain (JHS). MEP threshold is expressed as a percentage of the TMS output; the MEP latency is expressed in milliseconds (ms). The maximum amplitude of the MEP (MEPmax) is expressed as a proportion of the maximum motor response (Mmax). * denotes a significant difference relative to the other two groups

	NF (*n* = 30)	GJH (*n* = 29)	JHS (*n* = 20)	*p* value
**Slope (mV/%MSO)**	0.01 ± 0.01	0.01 ± 0.01	0.02 ± 0.02*	0.04
**MEPmax/Mmax**	0.39 ± 0.23	0.41 ± 0.27	0.49 ± 0.30	0.54
**MEP latency (ms)**	21.9 ± 3.4	21.3 ± 2.7	22.7 ± 3.5	0.33
**MEP threshold (%)**	61.7 ± 10.2	60.1 ± 14.1	62.4 ± 9.7	0.77

### Reflex responses

The slope of the H reflex recruitment curve and the Hmax/Mmax did not differ across the three groups (*p* = 0.32 and *p* = 0.12 respectively; see Table 4).

**Table 4:** Mean ± standard deviation of H reflex data from stimulation of the femoral nerve of three groups of participants; people with Normal Flexibility, no knee pain (NF), Generalised Joint Hypermobility, no knee pain (GJH) and Joint Hypermobility Syndrome, plus knee pain (JHS). Slope is expressed as amplitude in millivolts (mV) per stimulus output in milliamps (mA); the maximum amplitude of the H reflex (Hmax) is expressed as a proportion of the maximum motor response (Mmax)

	NF (*n* = 30)	GJH (*n* = 30)	JHS (*n* = 30)	*p* value
**Slope (mV/mA)**	3.50 ± 2.39	2.84 ± 2.12	2.77 ± 2.12	0.32
**Hmax/Mmax**	0.56 ± 0.23	0.44 ± 0.31	0.47 ± 0.29	0.12

## Discussion

This is the first study to explore corticospinal and spinal excitability in hypermobile people. We have demonstrated that one aspect of corticospinal control (excitability) was greater in JHS participants compared to people who have normal flexibility or asymptomatic GJH. That is, the slope of the MEP recruitment curve is steeper in the JHS group. All other variables of corticospinal and spinal excitability did not differ across the groups.

Our hypothesis was that people with symptomatic hypermobility would have reduced excitability of corticospinal control in comparison to people with asymptomatic hypermobility and people with normal flexibility. Therefore, the increase in the slope of the MEP recruitment curve was surprising and was the opposite to the hypothesised result. We had believed that the functional deficits and differences seen in proprioception [41–43] could be related to a reduction rather than increase in central excitability. However, this increase in slope could be due to the ability of the central nervous system to compensate for the lack of strength, afferent feedback and/or resultant functional instability that people with JHS demonstrate [10, 11, 40, 46] consequently, increasing excitability in order to impact recruitment of the motor neurone pool. Another factor that might influence this result is the pain, which was only being experienced by the participants with JHS. However, the impact of pain upon corticomotor control has been shown to be variable and may depend upon both the origin and duration of the pain as well as the function of the muscle [65–67]. It is therefore difficult to know if the pain here had an impact upon the slope of the recruitment curve and whether that change would impact proprioception.

Another difference that might influence this result is that the recordings were taken from the dominant leg of participants with NF and GJH, whereas the recording was taken from the most painful side of participants with JHS, which may or may not have been their dominant side. However, this is unlikely to have influenced the result as leg dominance has not been shown to impact corticospinal excitabilty [68].

In relation to a difference in afferent input, it is interesting to note that there were no differences in spinal reflex control. This negative result is important to report as it builds on the work of others and allows us to suggest alternative explanations as to why there could be differences in proprioception and co-ordination of movement [42, 52, 69] despite no deficits in this spinal control. Here, we evoked a monosynaptic spinal reflex by stimulating the peripheral nerve, i.e. proximal to the receptors within the muscle, thus excluding the sensory receptors’ contribution to the response. This suggests that one source of poor proprioception could be as a result of the receptor sitting within hypermobile connective tissue, which was not investigated here. An alteration to receptor output would not be surprising as it sits within tissue that has different extensibility and therefore altered dynamics [53, 54, 70] summarised by Palmer et al. [71]. It is therefore reasonable to assume that receptors which rely upon mechanical deformation to function, will have altered responsiveness, which in turn would alter afferent activity [72–75] but would not be picked up using the techniques here. Indeed, alteration to afferent input may relate to a perception of effort and central fatigue [76] that is commonly perceived by people with JHS [23].

The origin of dysfunction could have clinical significance. If the receptor rather than the central nervous system is the origin of proprioceptive loss, it suggests that building muscle tone to enable receptors to be more responsive may be an effective treatment strategy. Indeed, it is interesting to note that when hypermobile participants who demonstrated a delayed or absent long latency lower limb reflex were treated with a strength programme, this normalised this response [46]. This suggests that practicing reacting to perturbations and other ‘proprioceptive’ exercises might not be as useful as building muscle tone in order to improve proprioception.

There are some limitations to this study. Firstly, the ethnic diversity of the recruited participants does not represent the diversity of a London population. Seventy seven percent of the sample were of a white ethnic origin, whereas London’s population is approximately 60% white [77]. Promoting equality, diversity and inclusion in research is vital and this is particularly relevant here when hypermobility is thought to be more prevalent in some non-white populations. Greater focus on improved recruitment strategies is required to change this bias [78]. Secondly, it should be noted that some of the participants with JHS were taking neuromodulatory medication that precluded them from having TMS. This means that the investigation of excitability of the corticospinal response is not appropriately powered. However, even if the improved excitability of the corticospinal response is false, this doesn’t change the conclusion that poor ability to control movement is unlikely to originate from the central nervous system itself. Therefore, changing the environment in which the receptor sits, rather than focussing on rehabilitation that aims to excite the central nervous system may result in improvements to motor control.

In conclusion, bar an increase in slope of the quadriceps recruitment curve of people with anterior knee pain and JHS, there are no differences to corticospinal or reflex control measured here. This was surprising as poor proprioception is a factor in people with symptomatic hypermobility. The problem may relate to the pain however, it may relate to receptors sitting in hypermobile connective tissue. Strengthening to change the tone of muscle and consequently, the responsiveness of some receptors could influence proprioception and subsequently balance.

## Data Availability

The datasets used and/or analysed during the current study available from the corresponding author on reasonable request.

## Abbreviations

TMS: Transcranial Magnetic Stimulation
MEPs: Motor evoked potentials
H reflexes: Hoffman reflexes
M response: Motor response
GJH: Generalised Joint Hypermobility
JHS: Joint Hypermobility Syndrome
hEDS: Hypermobile Ehlers Danlos Syndrome
HSD: Hypermobility Spectrum Disorder
NF: Normal flexibility
VAS: Visual analogue scale
RF: Rectus Femoris
CED: Cambridge Electronic Design
MVC: Maximum voluntary isometric contraction
AMT: Active motor threshold
EMG: Electromyographic
Mmax: Maximal motor response
Hmax: Maximal H reflex response
MSO: Maximum stimulator output
HAP: Human Activity Profile
AAS: Adjusted Activity Score
